# Floor Identification Using Magnetic Field Data with Smartphone Sensors

**DOI:** 10.3390/s19112538

**Published:** 2019-06-03

**Authors:** Imran Ashraf, Soojung Hur, Muhammad Shafiq, Yongwan Park

**Affiliations:** Department of Information and Communication Engineering, Yeungnam University, Gyeongsan 38541, Korea; ashrafimran@live.com (I.A.); sjheo@ynu.ac.kr (S.H.); shafiq.pu@gmail.com (M.S.)

**Keywords:** floor identification, indoor localization, machine learning, smartphone sensors, geomagnetism, fingerprinting

## Abstract

Floor identification plays a key role in multi-story indoor positioning and localization systems. Current floor identification systems rely primarily on Wi-Fi signals and barometric pressure data. Barometric systems require installation of additional standalone sensors to perform floor identification. Wi-Fi systems, on the other hand, are vulnerable to the dynamic environment and adverse effects of path loss, shadowing, and multipath fading. In this paper, we take advantage of a pervasive magnetic field to compensate for the limitations of these systems. We employ smartphone sensors to make the proposed scheme infrastructure free and cost-effective. We use smartphone magnetic sensors to identify the floors in a multi-story building with improved accuracy. Floor identification is performed with user activities of *normal walking*, *call listening*, and *phone swinging*. Various machine learning techniques are leveraged to identify user activities. Extensive experiments are performed to evaluate the proposed magnetic-data-based floor identification scheme. Additionally, the impact of device heterogeneity on floor identification is investigated using Samsung Galaxy S8, LG G6, and LG G7 smartphones. Research results demonstrate that the magnetic floor identification outperforms barometric and Wi-Fi-enabled floor detection techniques. A floor change module is incorporated to further enhance the accuracy of floor identification.

## 1. Introduction

In recent years, Indoor Positioning and Localization (IPL) systems have received much attention from industry and academia because the proliferation of smartphones has opened new possibilities for indoor localization. IPL systems are grouped under two categories: infrastructure-based and infrastructure-free. Therein, the common examples of infrastructure-based technologies are Radio Frequency IDentification (RFID), Ultra-WideBand (UWB), Infra-Red (IR) and Global Positioning Systems (GPS) [1,2,3,4]. Fusion of these technologies is also reported in [5] where RFID is utilized along with cellular data. The frequencies from Ultra High-Frequency RFID (UHF RFID) and macro-cellular networks are used to refine the localization accuracy. The wide availability of cellular networks helps to calculate a coarse location which can be used by other localization technologies. Authors use the location calculated from RFID, and macro-cellular separately and fuse them to refine the location. However, the indoor localization is now shifting towards infrastructure-free technologies due to the ease of installation and cost effectiveness [6]. Towards this end, the Micro-Electro-Mechanical Systems (MEMS)-based built-in sensors of smartphones including accelerometers, magnetometers, and gyroscopes, etc. are of interest, which do not require any additional hardware to operate. We can find various applications of MEMS-sensors in most recent works [7,8,9].

The inception of Location-Based Services (LBS) also accelerated this adventure as precise location information serves the leading role in LBS. LBS such as navigation, assets tracking, shopping guide, etc. require a precise location for better user experience. LBS has special requirements including accuracy, availability, cost, and quality of positioning services which most IPLs are currently unable to achieve [10]. The emergence of long term evolution (LTE) helps to achieve better location accuracy in indoor environments. The small cell design is the basis for systems providing innovative location techniques to localize the user in medium/large indoor areas including malls, offices, and corporate buildings [11]; 5G networks will also help to refine the localization accuracy. Currently, localization systems mostly perform localization on the server side and location is sent back to the user. This causes communication delays and increases latency. With the increased data rate that 5G will offer, the latency can be improved in IPL. IPL can play a key role to improve the quality of 5G services as well. Accurate location information can be utilized by 5G services to improve Signal-to-Noise Ratio (SNR), mobility, scalability, and robustness, etc. [12].

Current IPL systems are not very attractive due to the single floor localization environment since a user can come across multiple floors during the localization process. This eventually reduces the accuracy of such systems and limits their wide applicability. In IPL systems, the accurate identification of the floor is very essential since it can play a vital role in reducing lifesaving efforts during emergency response actions. In general, the floor identification techniques can be categorized into three classes: purely Wi-Fi-enabled techniques, purely barometer-based techniques, and hybrid techniques. Wi-Fi-enabled techniques are the popular ones as they use an already deployed Wi-Fi network. Therein, they rely only on the signals from Wi-Fi Service Access Points (SAPs). However radio signals based floor identification (e.g., Wi-Fi, Bluetooth, RFID) is highly sensitive to building materials, wall separations, and floor plans and so they are vulnerable to random noise, path loss, multipath interference, shadowing, and so on [13]. Therefore, floors with an uneven structure and open spaces can cause high identification errors [14]. Floor detection using the cellular networks data has also been done in [15]. Floor detection, also termed vertical positioning, is calculated using the femtocells placed in the experiment area. The floor detection accuracies from global navigation satellite systems (GNNS), inertial sensors (INS), barometer and cell ID are compared. The results demonstrate that barometer performs better among all selected techniques and cell ID can work as a complementary technology. Dense deployment of cells is expected to introduce interference and reduce the accuracy.

The barometric pressure-based floor identification techniques collect data from a smartphone-embedded barometer sensor. For data collection, barometers are installed at reference positions at the floor(s) and are connected to a server so as to update the pressure readings periodically or on demand otherwise. However, such data can change over time very rapidly due to weather variations. For example, the data of barometric pressure vary approximately 1.68 hectopascals (hPa) in a short time of 15 min only [16]. The external atmosphere and internal conditions including window openings, air conditioning, and heating can compromise the accuracy of the system as well. Therefore, a more recent reference reading of the atmospheric pressure is required every time the floor identification is performed. The hybrid approaches make use of both Wi-Fi and barometer pressure sensors to increase the accuracy of IPL systems [9]. The fusion of these approaches is aimed to get fine floor identification results. However, such techniques show their dependence on the infrastructure as well. We can find the use of some other sensors including accelerometer and Bluetooth to achieve higher floor identification accuracy as in [17,18].

In this paper, we propose a floor identification mechanism based on the pervasive magnetic field data, which is an infrastructure-free solution. Magnetic field has been used in many research areas recently including indoor localization [19], building localization [20], and indoor-outdoor transition detection [21], etc. Inspired from such research works, we leverage it to identify specific floors. The proposed approach does not rely on wireless technologies like Wi-Fi and Bluetooth in order to avoid the side effects of path loss, shadowing, and multipath fading. Further, it is a cost-effective solution because it does not require additional sensors to be placed inside the buildings. It works on a natural phenomenon and is inspired by the indoor localization systems such as in [22,23]. Towards this end, we use the built-in magnetic sensors of smartphones in our proposed system. To the best of our knowledge, floor identification based on magnetic data is firstly proposed to compensate for the side effects of existing systems.

The key contributions of this research work can be summarized as follows:

- We propose a novel floor identification approach based on the pervasive magnetic field. The proposed approach does not need any assistance from Wi-Fi and barometers. It solely works with the magnetic sensor of the smartphone.
- We investigate the user phone carrying positions by a comparative analysis of machine learning techniques. Therein, three phone orientations, normal walking, call listening, and phone swinging, are identified with the help of smartphone accelerometer, gyroscope and magnetometer data. The proposed approach is reliable such that its accuracy is not affected by variations in the phone orientations or carrying positions of the mobile phone users.
- We suggest the floor change detection mechanism using features from the magnetic and accelerometer data. K Nearest Neighbor (KNN), Decision Trees (DT) and Naive Bayes (NB) classifiers are tested to discriminate user’s walk on stairs and a plain surface so as to augment the accuracy of floor identification.

The rest of this paper is organized as follows. Section 2 takes an overview of the related works. Section 3 narrates necessary details about the magnetic field. Section 4 describes the proposed methods for floor identification and stairs detection. Section 5 presents the experiment set up and discusses the results. Section 6 concludes the paper.

## 2. Related Work

The widespread growth of smartphones motivated many research works for indoor localization. A large body of work can be found in the literature which utilizes smartphone sensors including the magnetometer and inertial navigation systems [24,25,26]. Additionally, the fusion of various smartphone sensors has proven very fruitful for localization as in [22]. Authors use the fusion of magnetic and visual sensors to perform localization. They also make use of floor plans of a building to refine localization accuracy. Similarly, the embedded barometer sensor in smartphones has led to numerous research works related to floor detection. We only discuss the research works that utilize smartphone based sensors to determine the current floor of the users in a building.

Reseach works [16,27] explore Wi-Fi-based approach combined with the relative change in pressure to identify the current floor of the user. In [28], we can find another system that works on the fingerprint map built from barometer historic data using a crowdsourcing approach. In [29], another method based on Wi-Fi signals is suggested which identifies a floor by collecting labeled Wi-Fi fingerprints on one floor only. Therein, a graph embedding method is used on data from different floors in order to propagate labels. Later, training is performed to infer labels for unlabeled data. However, the fingerprint data is needed to be collected on all floors. It is only different from the traditional approach in a way that the data on one floor is labeled manually. The rest is labeled automatically using a machine learning technique. In [30], Locus is proposed for floor identification that takes into account the existing knowledge of infrastructure and Wi-Fi SAP (Server Access Points) using a heuristic approach. However, the accuracy of Locus system is limited to 95.3%. In [31], Wi-Fi-based floor identification method is proposed on a fingerprint database approach. The fingerprints are collected in a grid where each grid point is separated by 1.6 meters. Then a floor discriminate model is trained based on Fisher’s Linear Discriminant and selects *W* to maximize the ratio of between-class scatter and within-class scatter. The phone accelerometer is used to identify the user’s state of elevating up and down.

In [32], we can find the application of smartphone sensors. Therein, a Wi-Fi-based barometer sensor is placed on each floor. The reference data from each sensor is retrieved and updated on the SAP server. Whenever the floor identification is performed, reference barometric data is compared with the current data values of the smartphone. The experiments are performed using three different smartphones with ‘in-pocket’ and ‘out-of-pocket’ modes. However, the window of 5 seconds can only achieve an accuracy of 94.47%. Further, this approach requires reference data whenever the experiment is performed. It is infrastructure-dependent and always needs to communicate to the server while getting reference barometer pressure values. In [33], a KNN and Back Propagation Neural Network (BPNN)-based floor identification is proposed, which uses Wi-Fi-based information and smartphone sensors including accelerometer and barometer. BPNN system involves an offline phase to make a fingerprint database of available SAPs on each floor. During the online phase, K-NN and BPNN algorithms need to determine initial floor in order to load the database. Then, the floor detection is performed with the help of barometric pressure and accelerometer data.

In [34], a hybrid scheme called HYFI is proposed that uses data of Wi-Fi SAPs and that of a barometer on a floor. HYFI scheme works in two steps. The first step involves the calculation of the distribution probability of scanned SAPs during the offline phase and then utilizes Bayesian classification to identify the floor. The second step is used to compensate for the identification error caused by variable factors like hollow spaces and uneven floor areas on a floor. For this purpose, the floor information from the first step is used to initialize and calibrate barometer pressure-based floor identification. In [35], another floor recognition method utilizes Wi-Fi signals based on the classifier of the Support Vector Machines (SVM), in which data for each floor is collected using the smartphone. The fact that Wi-Fi signals have an obvious change when passed through walls and floors is used to discriminate the floors. In [36], another scheme is proposed that uses barometric pressure data to identify the floor of the user. The statistical features of Root Mean Square (RMS), kurtosis, skewness, peak-to-peak, crest factor, shape factor, margin factor and impulse factors are extracted from the barometer data. Then, a Fuzzy C-Means clustering algorithm is applied to the extracted features to identify various floors. The testing is performed on Indoor Positioning and Indoor Navigation (IPIN) 2016 competition data set for floor identification. In [37], a calibration-free probabilistic approach is used to identify the floor of the user.

However, Wi-Fi fingerprinting-based systems [29,31] require an offline phase of fingerprint collection which involves much of the time and cost. The major problem with the Wi-Fi fingerprinting is the periodic update to accommodate the Received Signal Strength Indicator (RSSI) changes over time. Other dynamic factors including the presence of obstacles also affect RSSI which ultimately reduces the overall accuracy of such systems. In [30], the heuristic-based approach is not generalized for different test beds and exhibits various accuracy results in various environments. In [31], it is pointed out that Wi-Fi fingerprint-based schemes use the Euclidean space, which is prone to error due to Received Signal Strength (RSS) from the neighboring floor. The neighboring floor may become very similar to the RSS from the neighboring room in many cases [31]. In the same way, the scanned RSS values for the same device may not be the same over time, even for a fixed location [38,39].

In barometer sensor-based systems, the data readings are required for a reference point of the barometric pressure. The altitudes are calculated with the barometric data and compared against the height of the buildings to get the floor information of the user. One option is to utilize the knowledge of user initial floor information and its barometer data but it reduces the usability of such systems. Towards this end, [28,40] rely on reference pressure value from barometer sensors deployed on each floor. In [41], only one reference barometer sensor is used. For this purpose, the height of each floor should be known. However, exact height information of each floor may not be available in many cases. Furthermore, this approach is not very practical where the floor structure is irregular. The fact that the barometer data has different readings caused by humidity and temperature further reduces the accuracy. In [40], another approach is used to fetch the reference barometric pressure of each floor where floor identification needs to be done by using those reference values to find the floor number. This approach is limited by the fact that the local authorities cannot provide the exact data of small areas or buildings frequently. Additional barometers at each floor are installed to overcome this issue but it incurs extra cost; not to mention the fact that such systems are infrastructure-dependent and cannot function without the installed reference sensors.

## 3. Overview of the Magnetic Field

The earth’s magnetic field is the omnipresent phenomenon caused by the convection currents in earth’s outer core. The magnitude of the earth magnetic field varies from 25 μ Tesla to 65 μ Tesla. During the last few years, magnetic field positioning has enticed an ample consideration for indoor localization primarily because of its pervasiveness and secondly due to its infrastructure independence. The earth magnetic field strength is uniform and is not commuted abruptly over a small area. However, the presence of ferromagnetic materials including steel-reinforced concrete, metallic doors, pillars or steel walls interfere with the natural magnetic field and introduce disturbances also called anomalies [26,42].

**Figure 1:**
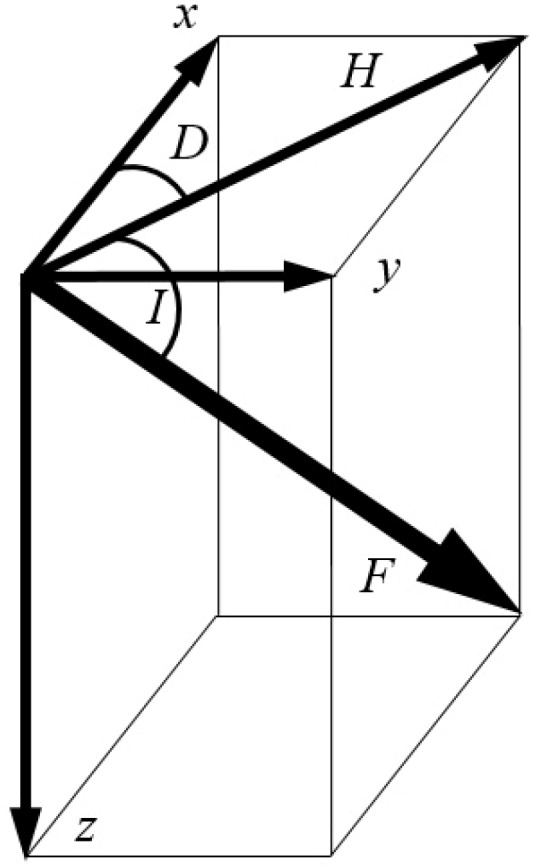
The components of magnetic field.

The magnetic field is comprised of seven components in total as shown in Figure 1. The *x*, *y*, and *z* show the magnetic field components for North, South and vertical intensities while *H* is the total horizontal intensity and *F* represents the total intensity or magnitude of the magnetic field at a given point. The *D* and *I* are measured in degrees and they represent the declination and inclination respectively.

**Figure 2:**
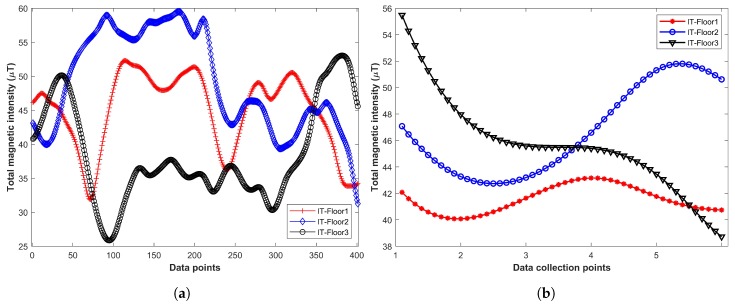
Total magnetic intensity, and its variation on same points of different floors. (**a**) Total magnetic intensity on different floors of IT building using S8, (**b**) Magnetic variation on same points of different floors using S8.

Magnetic field intensity varies in different buildings and exhibits a unique pattern that can be used as a fingerprint. Similarly, the magnetic field shows different patterns on different floors. Figure 2 shows the behanior of the magnetic field on different floors of IT building. Figure 2a shows the total magnetic intensity of IT buildings floors 1, 2, and 3. Figure 2b shows the magnetic samples collected on the same points at floor 1, 2 and 3 to show its variation. A total of 500 samples are collected at each point to view the magnetic variation on different floors. The stability of the magnetic field and its variant behavior on different floors make it suitable for floor detection.

**Figure 3:**
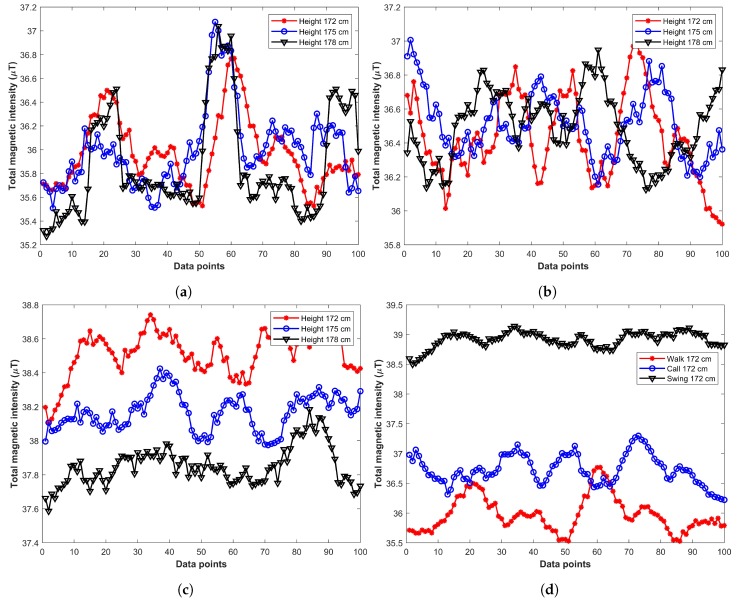
Impact of users’ different height and phone orientations on magnetic data. (**a**) Plain walk orientation with different heights, (**b**) Call listening orientation with different heights, (**c**) Phone swinging with different heights, (**d**) Same height different orientations.

One important aspect to investigate is whether the change in the magnetic value is caused by various orientations of the user or it is the height of the user that affects the magnetic value. We gathered magnetic data involving users of different heights including 172 cm, 175 cm, and 178 cm. The data was gathered for three phone orientations under study in this research. Figure 3 displays the effect of user’s heights, as well as, various phone orientations on the magnetic field. Figure 3a,b show that the impact of users’ height is very small for plain walk and call listening orientations. However, change in the magnetic value in phone swinging as shown in Figure 3c is slightly higher than those of the other two orientations. On the other hand, Figure 3d shows that different phone orientations cause a potential change in the magnetic field. So, the analysis revealed that the change in magnetic value is more caused by changing orientations than users’ heights.

**Figure 4:**
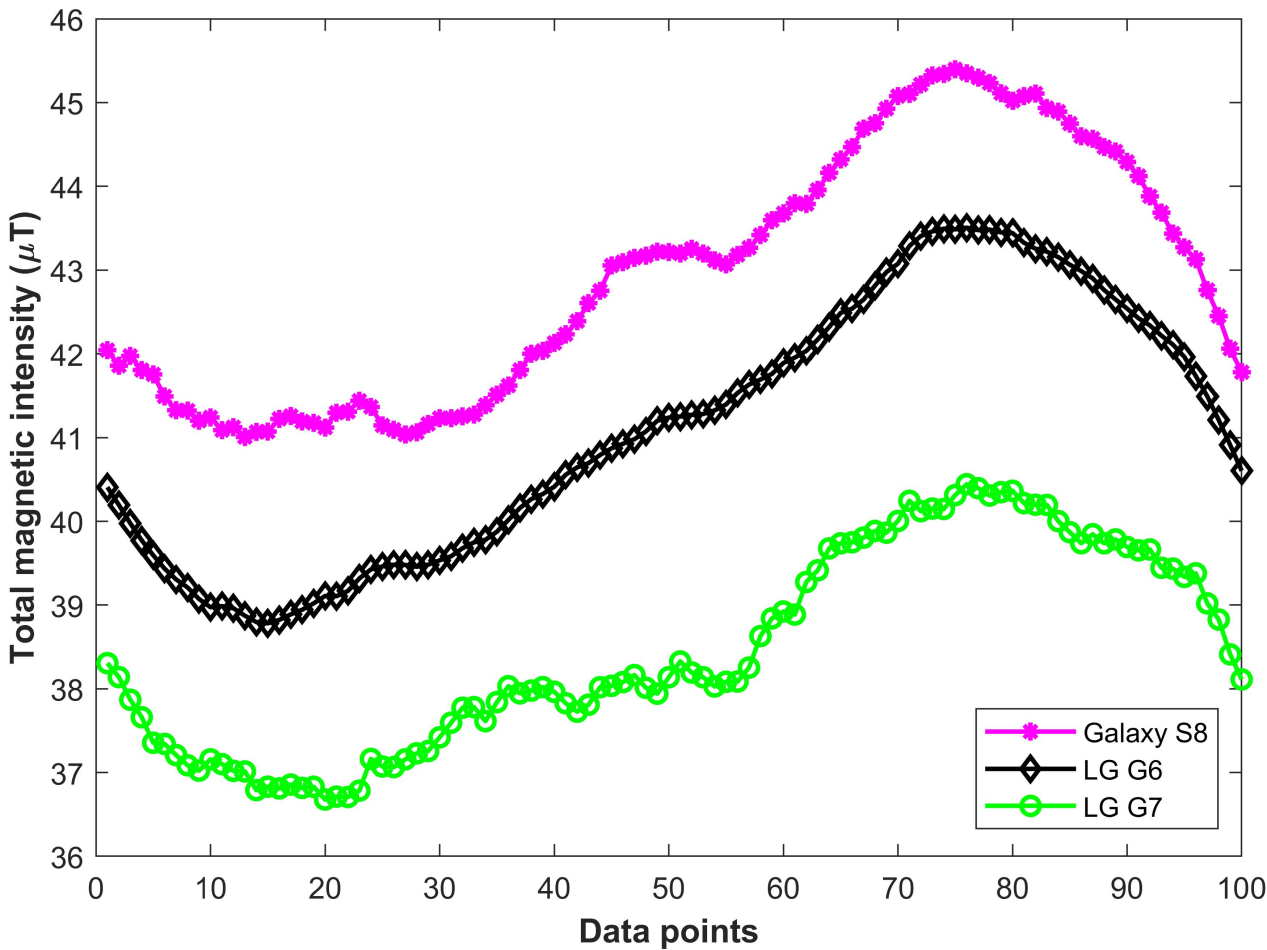
Impact of various smartphones on magnetic data.

The use of different smartphones also affect the magnetic data. Smartphones have magnetometers manufactured by various companies with different configurations. The magnetic data from different smartphones depend on the sensitivity and accuracy of the installed magnetometers. Figure 4 shows the magnetic data collected on floor 3 of IT building using Galaxy S8, LG G6, and LG G7.

**Figure 5:**
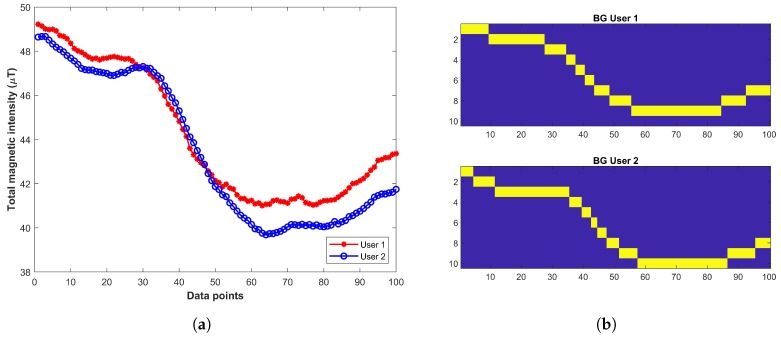
Magnetic data and its associated binary grid, (**a**) Data with same smartphone from different users, (**b**) Binary grid of magnetic data in Figure 5a.

Figure 5 shows the data collected from users of different heights and its associated Binary Grid (BG). We propose the use of Binary Grid (BG) approach to overcome the limitations of the user’s height. The BG approach is introduced in [7] to mitigate the impact of different smartphones. The approach does not use the magnetic intensity, instead, it transforms the magnetic data into patterns and compares those patterns. Figure 5b shows the impact of using the BG approach over magnetic intensity. Figure 5b shows that in spite of different magnetic intensity in Figure 5a, the generated BG are very similar and thus helps to reduce the impact of different smartphones and user heights.

## 4. Proposed System

The proposed system is composed of offline and online phases, which we, respectively, called calibration (or training) and floor identification phases. The offline phase involves surveying to collect the magnetic fingerprints of the area where floor identification is to be performed. Fingerprinting involves collecting multiple values at each selected position separated by a fixed distance and transforming the normalized values into magnetic fingerprints. The online phase incorporates data collected from user smartphone sensors to perform tasks of floor identification. Before we proceed, we summarize the used notations in Table 1.

**Figure 6:**
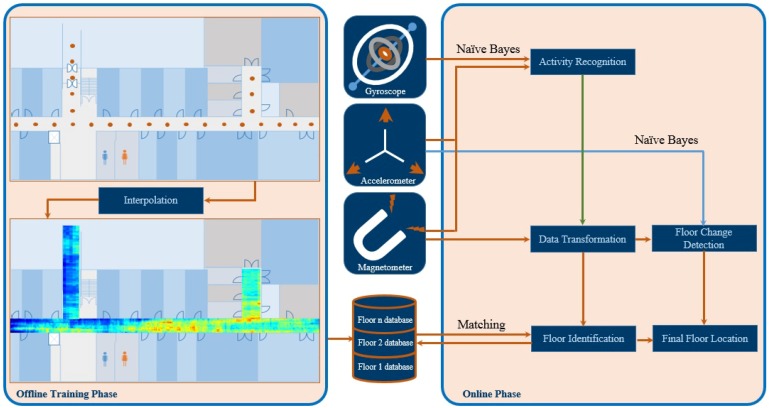
The block diagram of the proposed system.

Figure 6 presents the block diagram of the proposed method. The proposed method comprises three steps which we briefly explain in the following.

**Figure 7:**
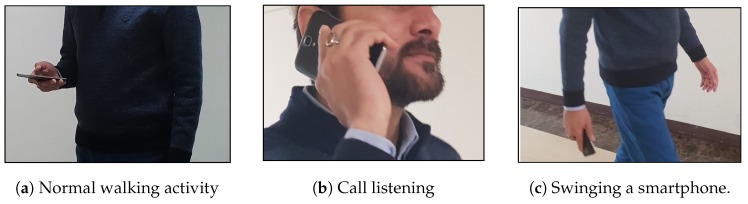
Types of user activities on a smartphone of the proposed system.

**Figure 8:**
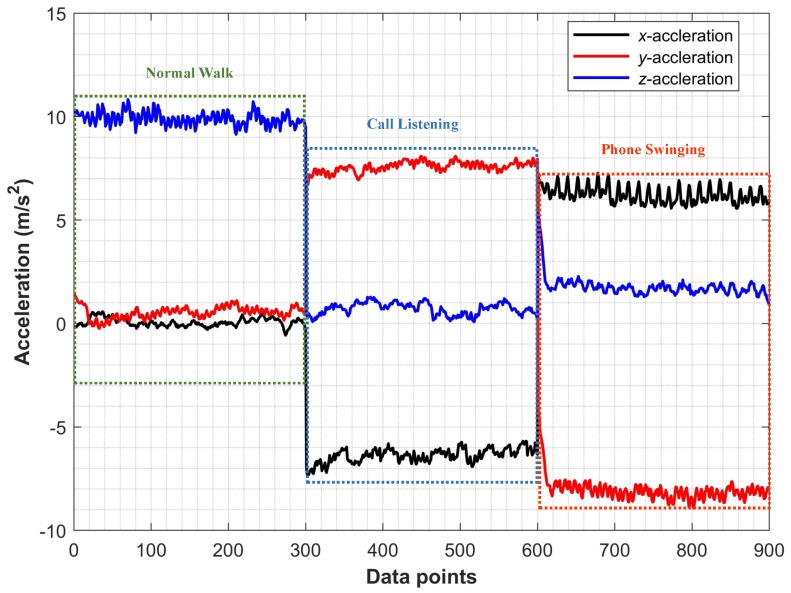
*x*, *y*, and *z* accelerations for user activities.

**Figure 9:**
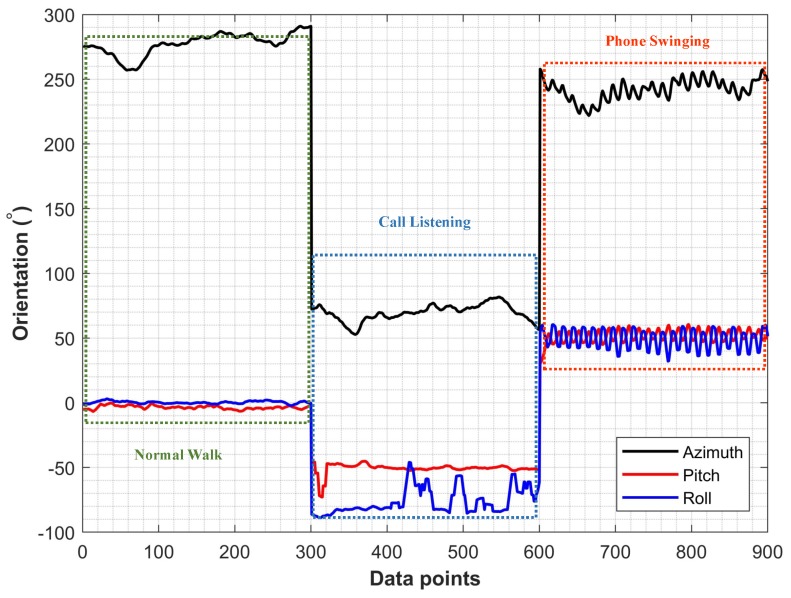
The phone orientation data for user activities.

**Step 1**: The first step determines the user activity. The user activities are distinguished as normal walking, call listening, and phone swinging as shown in Figure 7. In [43], we can find a neural network to classify these activities based on the acceleration data. However, use the orientation data from accelerometer and magnetometer sensors as well as the acceleration data to improve the accuracy in the real environment. The acceleration data of these user activities are different along *x*, *y*, and *z* axis as shown in Figure 8. The phone orientation by the user activities exhibits different behavior as well. Towards this end, we have shown the smartphone orientation data in Figure 9.

One can consider a variety of techniques to classify user activity, such as regression, deep learning, and machine learning techniques [44]. Deep learning models often require a substantial amount of data for training purposes to work in an unsupervised manner. Such models require additional time and computing resources for the training purposes. On the contrary, machine learning methods can work with a small amount of data labeled already. Therefore, we employ machine learning-based classification algorithms and analyze the performance of KNN, DT, and NB. The selected classifiers classify phone carrying position of the user so that the collected magnetic data can be transformed according to the user smartphone carrying position. However, it is not a trivial task since the accuracy of the floor detection algorithm heavily depends on it. If the magnetic data transformation is not good, it may generate wrong results. DT based classification is one of the most widely used techniques in machine learning. It can imitate human decision making and deduce decisions based on the provided feature set. A decision tree consists of a root node, internal node, and leaf. A node is a representation of a feature while the edge is associated with the decision or rule [45,46]. The gain ratio is the most commonly used criteria for tree split. DT are propitious on account of their easy interpretation and being computationally inexpensive. In addition, they are non-parametric and do not need prior assumptions about the feature set.

KNN is another widely used classifier also called the lazy learner. It is also a non-parametric technique and holds no assumptions on the data distribution. It draws the boundary lines between the classes of data based on the distance between the data points. Euclidean distance is the most commonly used distance measure in KNN along with many others including Mahalanobis, City block, Manhattan distance, etc. A new object is classified on the basis of its distance to a particular class [47]. Despite the fact that it is simple and easy to understand, it can become slow if the training data is large [48]. The NB classifier can predict the probability of a given sample to a particular class based on the Bayesian theorem. NB is simple yet can outperform other sophisticated classification methods [49,50]. NB assumption of attributes independence results as,

(1) $$P\left(X|C_{i}\right)\approx \prod_{k=1}^{n}P\left(x_{k}|C_{i}\right)$$

which means that for a given sample *x*, the class $C_{j}$ is associated with the sample which achieves the highest posterior probability which is calculated by,

(2) $$P\left(C_{i}|X\right)=\frac{P\left(X|C_{i}\right)P\left(C_{i}\right)}{P\left(X\right)}$$

where *X* is the set of training samples while *C* denotes the set of class labels.

The features from smartphone accelerometer, gyroscope and magnetometer are used to identify user activities. The accelerometer provides acceleration in *x*, *y*, and *z* directions. The total acceleration, *a*, can be calculated as follows,

(3) $$a=\sqrt{a_{x}^{2}+a_{y}^{2}+a_{z}^{2}}$$

However, the acceleration has error called bias, even after the calibration. This bias needs to be estimated and removed to get accurate distance information. If the smartphone is put motionless on a plain surface, the acceleration in *x* and *y* direction should be 0 and *z* acceleration should be 1 g (9.81 m/s^2^). So, any additional reading is marked as bias. We have calculated the bias-free acceleration in the following,

(4) $$a_{x}^{c}=a_{x}^{m}-S*a_{x}^{a}$$

where $a_{x}^{c}$, and $a_{x}^{m}$ represent the measured *x*-axis acceleration while $a_{x}^{a}$ is the actual *x*-axis acceleration. The total bias-free acceleration for a given time *t* can be calculated as,

(5) $$a_{t}^{c}=\sqrt{{a_{xt}^{c}}^{2}+{a_{yt}^{c}}^{2}+{a_{zt}^{c}}^{2}}$$

The features from corrected acceleration along with gyroscope and magnetometer are used to classify a particular user activity after the acceleration values are corrected.

**Step 2**: We here conduct floor identification process by Algorithm 1. We investigate the use of magnetic field data for floor identification. The first task is to transform the magnetic data with respect to the identified phone orientation. The magnetic fingerprint database is built relative to the global coordinates irrespective of the phone orientation. The fingerprinting involves surveying for data collection that requires time, cost, maintenance and updating as well. However, updating is not required unless any major changes involving ferromagnetic materials in internal infrastructure are made since the magnetic field is very stable. The first important task to perform floor identification is to get a fingerprint database of each floor of a building of interest.
**Algorithm 1** Floor Identification Using Magnetic Data1:Input: magnetic data *M*, User phone orientation *O*2:**for**i⟵1to5**do**3: $S_{m}⟵transformMagData\left(O_{i},M_{i}\right);$4: **for** $j⟵N_{b}$ **do**5:  $E_{d}⟵calEucDistance\left(S_{m},DB_{bj}\right);$6: **end for**7: $F_{c}⟵argmin\left(E_{d}\right);$8:**end for**9:$F_{b}⟵removeOutliers\left(F_{c}\right);$

**Figure 10:**
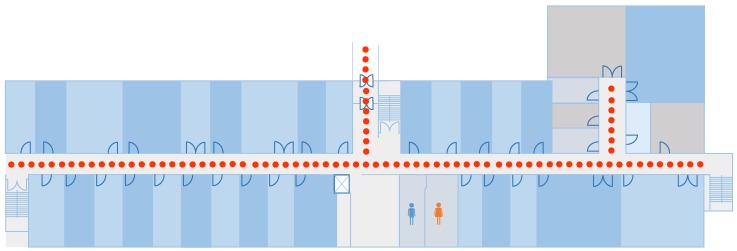
The magnetic fingerprint database making process.

**Figure 11:**
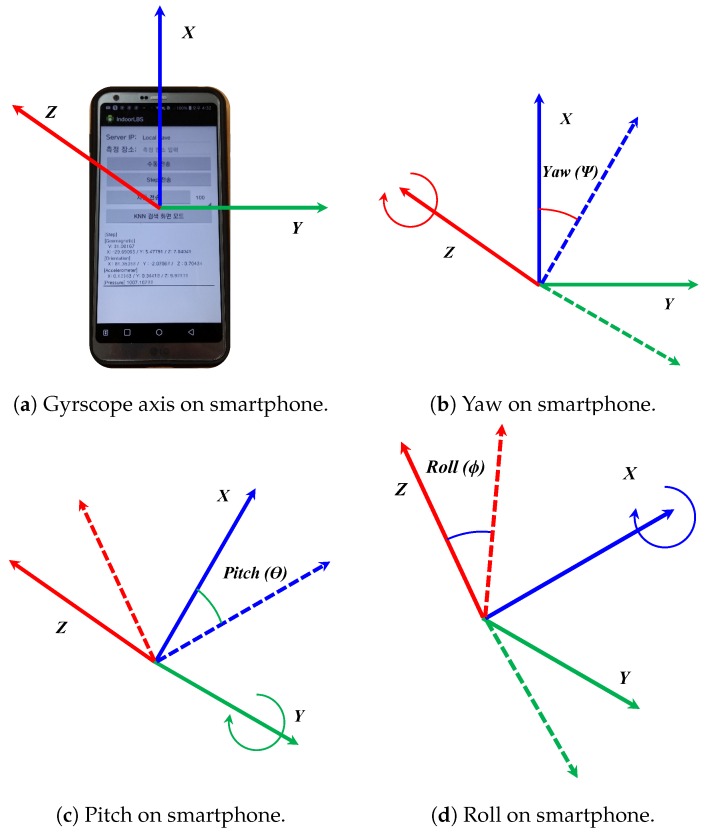
The yaw, pitch, and roll on a smartphone.

The data collection points are separated by 1 m as shown in Figure 10. This shows the example case of making the fingerprint database at IT building floor 3. A total of 50 magnetic samples are collected at each point with a sampling frequency of 10 Hz. The collected samples are averaged and later a spline interpolation is performed to generate the intermittent values between the given points. The generated values are then transformed into magnetic patterns using the BG algorithm proposed in [7]. The transformed patterns serve as the fingerprint database. We now can transform the magnetic data since the database is built with respect to global coordinates. We use two coordinate systems called global coordinates, and body coordinates. Global coordinates represent the Earth-fixed coordinate system while the smartphone coordinates are taken as body coordinates. The magnetic data are transformed with respect to the phone orientation to match it against the database. Let $M_{G}$ be the magnetic field value at the global coordinate system and $M_{B}$ be the magnetic readings at the smartphone coordinate system. The relationship between $M_{G}$ and $M_{B}$ can be defined as [51],

(6) $$M_{B}=R_{x}\left(\phi \right)R_{y}\left(\theta \right)R_{z}\left(\psi \right)M_{G}$$

where $R_{x}\left(\phi \right)$, $R_{y}\left(\theta \right)$, and $R_{z}\left(\psi \right)$ are the corresponding matrices for yaw, pitch, and roll. Yaw, pitch, and roll respectively represent rotation around *z*, *y*, and *x* axis as shown in Figure 11.

We need to find yaw, pitch, and roll to transform the magnetic data. Yaw is the rotation around *z*-axis of the body represented by ψ. The rotation matrix $R_{z}$ is represented as,

(7) $$\begin{array}{c} R_{z}\left(\psi \right)=\left[\begin{array}{ccc} cos(\psi ) & sin(\psi ) & 0\\ -sin(\psi ) & cos(\psi ) & 0\\ 0 & 0 & 1 \end{array}\right] \end{array}$$

Pitch represents the rotation around *y*-axis of the body, denoted by θ, which is calculated as,

(8) $$\begin{array}{c} R_{y}\left(\theta \right)=\left[\begin{array}{ccc} cos(\theta ) & 0 & -sin(\theta )\\ 0 & 1 & 0\\ sin(\theta ) & 0 & cos(\theta ) \end{array}\right] \end{array}$$

Roll is the rotation around *x*-axis of the body, which is denoted by ϕ and is calculated by,

(9) $$\begin{array}{c} R_{x}\left(\phi \right)=\left[\begin{array}{ccc} 1 & 0 & 1\\ 0 & cos(\phi ) & sin(\phi )\\ 0 & -sin(\phi ) & cos(\phi ) \end{array}\right] \end{array}$$

Once yaw, pitch, and roll are calculated, the transformation of magnetic data can simply be done as Equation (6). The magnetic sample, $S_{m}$, is matched against the databases of all floors of a building to calculate the Euclidean distance,

(10) $$E_{d}=\sqrt{{\left(S_{m_{i}}-DB_{bj}\right)}^{2}}.$$

The Euclidean distance is calculated for each floor database. The term $DB_{bj}$ indicates the databases of a building *b*. Since there are multiple floors in buildings, so *j* refers to floor number in building *b*. Equation (10) shows that the magnetic sample is matched against the database of each floor in a building to calculate the Euclidean distance. The floor with the minimum Euclidean distance is the floor candidate for a given magnetic frame. The process is repeated for the five frames of data. The ‘*frame*’ indicates the data collected over 1 s from smartphone sensors. The data are collected at a sampling rate of 10 Hz (a new value after every 100 ms). We have used five frames based on the empirical findings. Our experiments reveal that if we use less than five frames, floor detection accuracy is affected. Using more frames of data surely increases the accuracy, yet, longer data may not be available in many situations especially when the indoor space is short. The processing of five frames yields five potential candidates (one for each frame). Hence, each frame has one vote for the final decision. The final decision is made against the majority of the votes.

Once the floor identification is done using five frames of the magnetic data, it produces a set of five predictions. So, it is possible that the predictions are not consistent every time. The prediction set may contain predictions for different floors even though the user is at the same floor. We found that the prediction set can have one of the following forms: $F_{c}=\left\{1,1,1,1,3\right\}$ or $F_{c}=\left\{1,1,2,1,3\right\}$ $F_{b}$ is floor 1. $F_{c}=\left\{1,1,2,2,3\right\}$ or $F_{c}=\left\{1,1,3,2,3\right\}$ or $F_{c}=\left\{1,2,3,4,5\right\}$ $F_{b}$. Where each element of a set is a floor prediction made using one frame of the magnetic data. The floor with a higher prediction frequency is regarded as the predicted floor. However, if the prediction frequency of multiple floors is same in the prediction set, the decision is made by,

(11) $$F_{b}=argmin\left(E_{d}\left(F_{c}\right)\right).$$

**Step 3**: This step helps in identifying the floor change when the user walks on the stairs. The task of floor change detection is achieved with the help of accelerometer and magnetometer data. The change in acceleration magnitude and acceleration patterns are different when the user walks on the stairs than that of walking on a plain floor. We only need to identify the important features that can be used to determine the user state of walking on the plain floor and stairs. We have identified six features in the accelerometer data that are suitable to discriminate the above-mentioned states.

The selected features are based on the output of the Recursive Feature Elimination (RFE) method [50]. It is a famous method to perform the important task of feature selection. It can fit a model and remove the weakest feature(s). The features are ranked by their importance by recursively eliminating feature(s) at each iteration. The optimal number of features are found by using cross-validation with RFE method. RFE features ranking is given in Table 2 while the features selected after RFE and their notations are discussed in Table 3.

**Figure 12:**
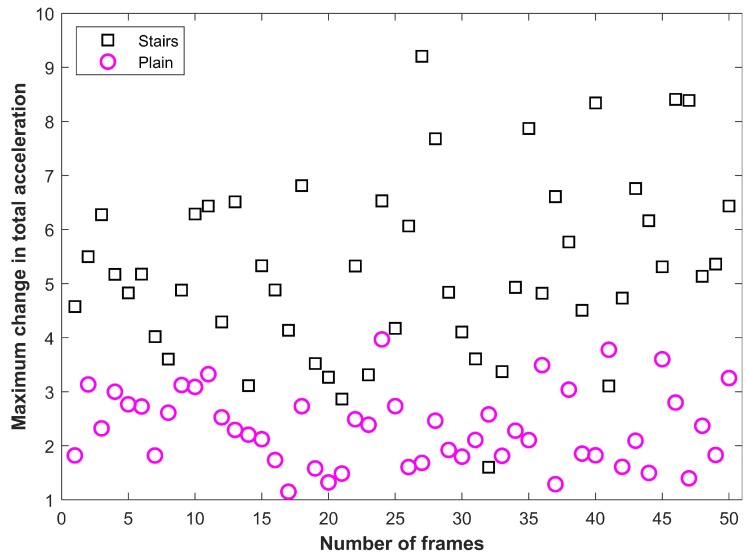
Peak values in total acceleation.

**Figure 13:**
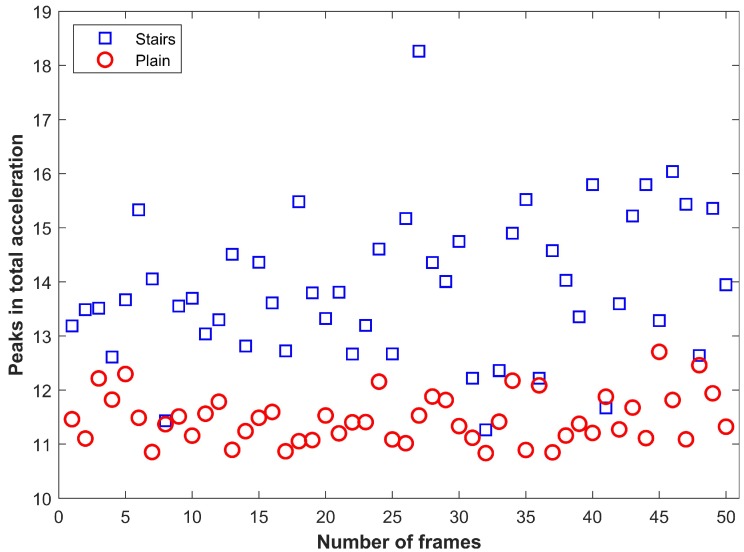
Maximum change in total acceleration.

The selected features show different values for users state of walking on stairs and plain floor. For example, Figure 12 shows the selected feature of ${⋁}_{a}$ for a user walking on the plain floor and stairs. Figure 13 shows the feature of ${⋁}_{\Delta_{a}}$ for the given user activities. It can be seen that the magnitude of these features is very much different for two user activities. In Figure 12 and Figure 13, each displayed value is calculated for 1 frame of data at a sampling frequency of 10 Hz. The selected features are fed into the machine learning techniques including KNN, DT, and NB to train the model. Later, the trained model is used to detect the floor change using the user collected data. During Step 2, when floor identification is performed, we do not track the user’s floor. The normal process involves the identification of a specific floor every time we get the data from the user smartphone to further enhance the accuracy of the proposed method.

The complete process of floor identification runs on the server side. The server is an Intel i7, 3.20 GHz running with 16.0 GB installed memory. Floor identification takes 0.039 s on average for one floor identification request. The processing time is averaged for a total of 24,000 floor identification requests from three smartphones Galaxy S8, LG G6, and LG G7.

## 5. Experiment and Results

We here describe the experiment set up before we discuss the experiment results. The location of the experiment and the details of the buildings are described as well.

### 5.1. Experiment Setup

**Figure 14:**
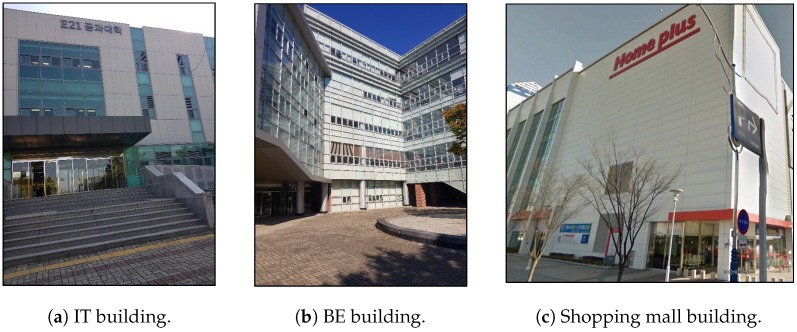
Buildings used in the experiment.

The experiments are carried out at two different places including a university campus The Yeungnam University Campus is located in Gyeongsan, North Gyeongsang, South Korea. and a shopping mall Home plus supermarket is located in Gyeongsangbuk-do, Gyeongsan-si, South Korea. More precisely, we have selected two buildings including the Information Technology (IT) department building and Business & Economics (BE) department building from the university campus. The IT building has 3 floors while the BE building has 5 floors in total with 1 underground floor. The shopping mall has 6 floors in total including 2 underground floors. Three buildings where the experiment has been performed are shown in Figure 14. The data readings at the buildings, selected for the experiment purpose, are collected during different times of the experiment period. The magnetic data are collected over a period of 6 months to conduct the experiments for floor identification. Samsung Galaxy S8 (SM-G950N) is used for the data collection purpose. The details of the sensors used for the data collection are summarized in Table 4. The data are collected over a sampling rate of 10 Hz (a new data sample after every 100 ms) for magnetometer, accelerometer, gyroscope, and barometer. The Wi-Fi data is collected with a sampling rate of 1 Hz.

### 5.2. User Phone Carrying Position

**Figure 15:**
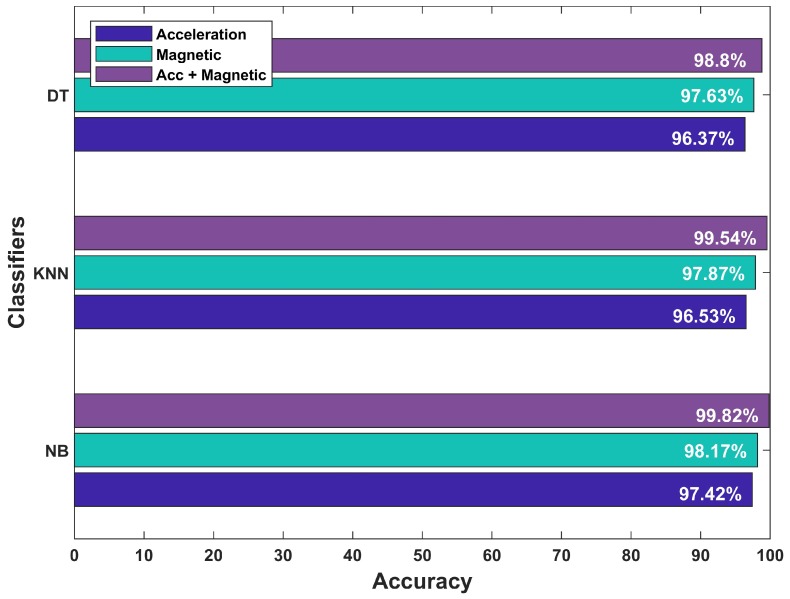
Results for phone orientation classification.

We used the third floor of IT building for the training data of user phone carrying positions. The training data contains 900 samples of each orientation which are fed into the selected classifiers. Supervised training is used where the labeled data is used for the training. The training vector contains the magnetic azimuth, pitch, and roll and acceleration in *x*, *y*, and *z* directions. During the testing, the data from three buildings are used and 8000 samples of each phone orientation are tested. Figure 15 shows the average results for three phone orientations. The accuracy of NB classifier is observed higher than that of the DT and KNN. Machine learning-based classifiers show good performance to classify user various phone holding positions. The accuracy of the acceleration data remains lower than that of the magnetic data for all classifiers since the acceleration data is noisy compared to the magnetic data. We achieved higher accuracy when the acceleration and magnetic data are combined.

### 5.3. Floor Identification

Floor identification algorithm is used on the magnetic data for floor identification. The accuracy of the proposed method is compared to the Barometric data and Wi-Fi data approaches. We have used the same smartphone to collect the Wi-Fi, magnetic and barometer data. The hypsometric equation [55] is used to perform barometric floor identification as follows,

(12) $$h=\frac{{(\left(\frac{P_{o}}{P}\right)}^{\frac{1}{5.257}}-1)\times \left(T+273.15\right)}{0.0065}.$$

where *P* is the current atmospheric pressure, $P_{o}$ is the sea-level pressure which is 1013.25 hPa, *h* is the altitude, and *T* is the temperature. We have used *T* as 15 °C in Equation (12).

For a floor, we calculated the reference barometric pressure (denoted by RBP) of 15 s data. This process is repeated for every floor where we perform the experiment. The RBP is used to calculate the reference altitude (denoted by RA) of the associated floor. The calculated RA is for normal walking mode. Now, we need to define the altitude restriction (denoted by AR) for call listening, and phone swinging mode. During the normal walking mode, the smartphone is held at an altitude of 1 m from the ground floor. So, we define AR as RA±1 m for call listening, and swinging mode, respectively. The value of RBP is calculated every time we perform the experiment since atmospheric pressure changes during the different times of the day. The barometric data for 1 s is averaged to calculate the altitude. The calculated altitudes are compared with RAs to find the floor. On the other hand, the Wi-Fi floor detection is performed with the fingerprint database approach. Wi-Fi data are collected at specified points separated by 1 m. The fingerprint database contains Basic Service Set Identifier (BSSID) and RSSI in dB of each scanned SAP on that point. Two elements used to perform floor identification with Wi-Fi are Highest RSSI of specific SAP’s at individual floors and Matched RSSI’s of scanned SAPs with the database.

**Figure 16:**
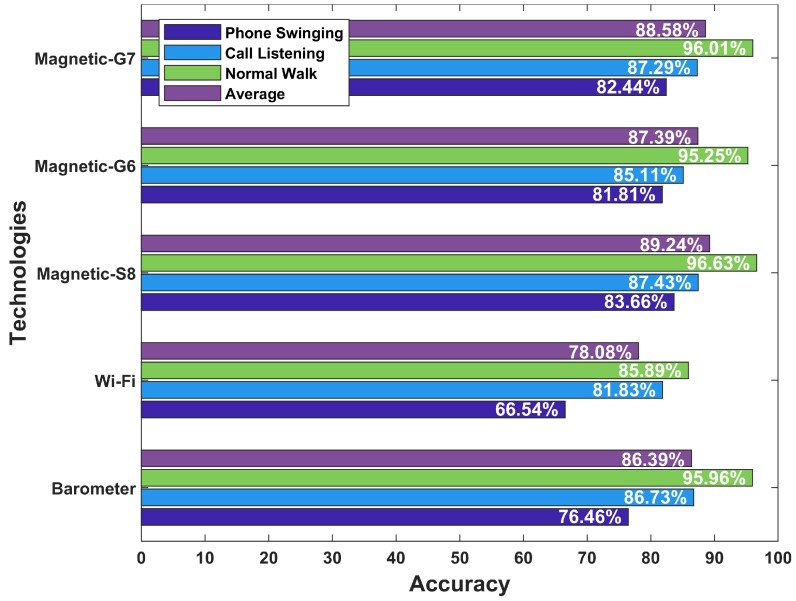
Results for floor detection.

During the testing process, the BSSID of scanned SAP and its associated RSSI value is compared with the fingerprint database of each floor. The fingerprint database of a floor with lowest error is the most probable floor of the user. The error is calculated based on two criteria that are the number of matched SAPs and the lowest difference per matched SAP. Experiments are performed within specified buildings for 3 different smartphone orientations. The accuracy results are shown in Figure 16, in which the barometer achieves an overall accuracy of 86.39%. The floor identification accuracy using the barometer is higher for the normal walking mode and is lowest when the user walks with phone swinging. The overall accuracy is high because the reference value is used every time the floor detection is performed. However, the major limitation of using the barometer is the retrieval of reference barometric pressure or altitude when floor identification is needed. Normally, it is achieved by placing a barometer sensor at each floor at a specific height from the ground and then use the reading from that sensor to do the floor detection. So, a recent dynamic reference reading from barometer increases floor detection accuracy with the barometer. Another option is to place one sensor at only one floor of a building and use the calculated altitude as the reference altitude. Other altitude values of the floors are derived using the reference altitude and known heights of each floor. However, this approach is not as accurate as using the reference data from each floor.

We observe that Wi-Fi-based floor identification has a lower accuracy compared to that of the magnetic and barometric floor identification as shown in Figure 16. Wi-Fi floor detection accuracy highly depends upon the matching of a higher number of SAPs and similarities in RSSI values. BE building has 8–10 SAPs on average. On the other hand, IT building has an average of 30 SAPs at each scanning position. So, the overall accuracy is respectively observed as 90.83% and 77.58% for the normal walking mode. Similarly, the number of SAPs in the shopping mall are high and so the floor detection accuracy in the shopping mall is observed as 87.76%. The average accuracy for Wi-Fi-based floor identification is 78.08% where the average accuracy is calculated as:

(13) $$Average=\frac{C_{p_{nw}}+C_{p_{cl}}+C_{p_{ps}}}{T_{p_{nw}}+T_{p_{cl}}+T_{p_{ps}}}\times 100$$

where $C_{p}$, and $T_{p}$ represent correct and total predictions for different orientations.

The factor which limits Wi-Fi performance is its volatile behavior. Wi-Fi signals are affected by many dynamic factors including the presence of people and other obstacles at various orientations and direction of the smartphone during the scanning process. Furthermore, Wi-Fi signals are depleted over time and so the accuracy of the system goes down.

Experiments for magnetic floor detection are done with Galaxy S8, LG G6, and LG G7 smartphones. The magnetic database is built using Galaxy S8 while experiments involve three smartphones. The magnetic database does not contain magnetic values. Instead it stores the magnetic patterns formed using the BG approach.

Three smartphones have different results for magnetic floor detection. As described in Section 3, the magnetic data is affected when different smartphones are used. The effect of smartphone heterogeneity lead to different floor identification results. Although we have used BG, even so, the magnetic patterns from different smartphones are not exactly the same. It results in different accuracies for different smartphones. However, the experiments results demonstrate that the magnetic floor identification is feasible and more precise than that of Wi-Fi.

The experiment results prove that the magnetic data has a huge potential in this regard as shown in Figure 16. The average accuracy with S8 is observed as 89.24% which is higher than that of the barometer data (i.e., 86.39%). Although the magnetic floor identification with G6, and G7 are 87.39%, and 88.58% respectively, yet, it is still better than those of barometer and Wi-Fi. Additionally, unlike the barometer floor identification where we retrieved a reference reading every time, the magnetic data are collected only once to make the fingerprint database. The magnetic field data shows stability over time. Moreover, the use of magnetic patterns mitigate the impact of device heterogeneity and users heights. The magnetic field is pervasive and does not require any additional sensors to be installed like the barometric floor detection does. The data are collected using a smartphone built-in magnetic sensor to perform the floor identification. The individual accuracy for normal walking, call listening, and phone swinging modes are also good for magnetic floor identification. Therefore, it is safe to say that magnetic floor detection is robust and energy efficient compared to the Wi-Fi-enabled systems.

### 5.4. Floor Change Detection

**Figure 17:**
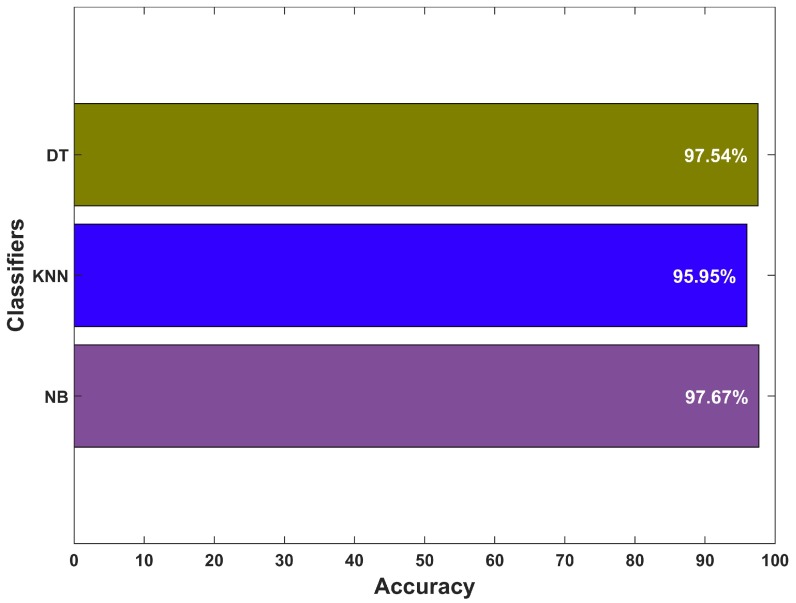
Results for floor change detection.

We take the collected data and perform the floor detection for each user request without the knowledge of his prior floor. Since the floor of the user during the floor identification process is not tracked. This process does not include the stairs detection. The floor change detection step is added to refine the floor identification accuracy. The results of floor change detection using the above mentioned machine learning methods are shown in Figure 17. Therein, all selected classifiers perform well in detecting the stairs using the accelerometer and magnetometer data features. However, NB outperforms KNN and DT classifiers. Therefore, we use NB in our proposed floor identification method to improve accuracy. The accuracy can further be improved with the help of the floor change detection method. The improvement is due to the fact that the user is not walking on stairs very frequently and use them when he/she needs to change the floor. The average accuracy of the barometer, and Wi-Fi-based floor identification after adopting the floor change detection method is observed as 88.12%, and 79.64%, respectively. Similarly, the magnetic floor identification sing Galaxy S8, LG G6, and LG G7 can further be increased to 91.02%, 88.93%, and 90.31%, respectively with floor change detection module. The magnetic field based floor detection has the highest accuracy among the selected techniques.

## 6. Conclusions

Modern indoor localization systems work in a single floor environment, in which floor information is taken as a priori. Wi-Fi and barometer data are traditionally used to determine the user floor inside a building, which is infrastructure-dependent and error-prone due to dynamic changes in the environment. Such assumptions are not applicable to real-world scenarios. In this study, we present a novel approach to determine the floor of a building using the pervasive magnetic field data. The earth’s magnetic field is a natural phenomenon and can be leveraged for the said task easily. The magnetic field is very stable and reliable. Moreover, it does not need any additional sensors and can be measured with the help of a simple magnetometer present in all modern smartphones. The proposed approach first utilizes the accelerometer and magnetic data to identify the user phone orientation. Towards this end, a Naïve Bayes classifier is used whose accuracy is observed as 99.82% for *normal walking*, *call listening* and *phone swinging* modes. The magnetic data is transformed to perform the floor identification task when a specific phone orientation is identified. The magnetic datapoints of 5 s are utilized to identify the current floor of the user. It works with the magnetic patterns database built during the offline phase of the system. The experiments are performed in two buildings of a university campus and a shopping mall. The performance of magnetic field data-based floor identification is compared with Wi-Fi and barometer-based floor identification systems. Our results reveal that the magnetic field-based floor identification approach outperforms both of the existing techniques. Experiments are performed using Samsung Galaxy S8, LG G6, and LG G7 to investigate the impact of device heterogeneity. The individual accuracy of S8, G6, and G7 is 89.24%, 87.39%, and 88.58%, respectively, using the magnetic data. The average accuracy of the barometer, W-Fi, and magnetic techniques can, respectively, further be increased to 88.12%, 79.64%, 91.02%, 88.93%, and 90.31% if floor change detection module is used. Current floor detection considers only three of the user phone orientations. In real scenarios, the user phone movements are of a more complex nature. We intend to add more orientations in our future work.

## Figures and Tables

**Table 1:** Summary of the notations used in Algorithm 1.

Notation	Description of Notation
*N*	Total number of floors in a building.
*O*	User phone orientation.
*M*	Magnetic data from smartphone.
$S_{m}$	Transformed magnetic sample.
$E_{d}$	Set of Euclidean distance between magnetic sample & fingerprint database.
$DB_{b}$	Fingerprint magnetic database of a floor in building *b*.
$F_{c}$	Set of calculate floor candidates.
$F_{b}$	Identified floor where the user is right now.

**Table 2:** Features’ ranking using RFE.

Feature Name	Feature Ranking	Selected
Average total magnetic intensity per frame	11	False
Average magnetic *x* intensity per frame	8	False
Average magnetic *y* intensity per frame	9	False
Average change in total magnetic intensity per frame	7	False
Maximum change in total magnetic intensity per frame	4	True
Average *x*-acceleration per frame	3	True
Average *y*-acceleration per frame	5	True
Average *z*-acceleration per frame	10	False
Average total acceleration per frame	2	True
Peak values in total acceleration per frame	1	True
Maximum change in total acceleration per frame	6	True

**Table 3:** Summary of the features selected after RFE.

Notation	Description of Notation
${\bar{a}}_{x}$	Average *x*-acceleration per frame.
${\bar{a}}_{y}$	Average *y*-acceleration per frame.
$\bar{a}$	Average total acceleration per frame.
${⋁}_{a}$	Peak values in total acceleration per frame.
${⋁}_{\Delta_{a}}$	Maximum change in total acceleration per frame.
${⋁}_{\Delta_{Mf}}$	Maximum change in total magnetic intensity per frame.

**Table 4:** List of the sensors used for the experiment.

Item	Description
SM-G950N	Galaxy S8 Octa-core, Adreno 540 GPU, Android 7.0, 4 GB RAM
Magnetometer (AK09916C)	3-axis, 16-bit, sensitivity 0.15 μT/LSB, temperature −30 +85 °C, 1.1 mA @ 100 Hz [52]
Accelerometer (LSM6DSL)	3-axis, 16-bit, sensitivity 0.061 mg/LSB, Temperature −40 +85 °C, 0.29 mA @ 52 Hz [53]
Gyroscope (LSM6DSL)	3-axis, 16-bit, sensitivity 125 mdps/LSB, Temperature −40 +85 °C, 0.29 mA @ 52 Hz
Barometer (LPS22HB)	24-bit, 260 1260 hPa range, temperature −40 +85 °C, 3 μA
Wi-Fi (Broadcom BCM4361)	Single-chip dual-band 3-stream 802.11ac, 1.3 Gb/s, 256-QAM modulation, LDPC (Low Density Parity Check) codes [54]
